# Her2 Status Discrepancy Between Core Needle Biopsy and Surgically Resected Mastectomy Specimen: A Clinical Case

**DOI:** 10.7759/cureus.33501

**Published:** 2023-01-08

**Authors:** Ana Carolina Vasques, Mafalda Miranda Baleiras, Marta Pinto, Filipa Ferreira, Ana Martins

**Affiliations:** 1 Medical Oncology, Hospital São Francisco Xavier, Lisbon, PRT; 2 Medical Oncology, Centro Hospitalar de Lisboa Ocidental, Lisbon, PRT

**Keywords:** intratumoral heterogeneity, concordance, biomarker, her2, breast cancer

## Abstract

The biomarker concordance between core needle biopsy (CNB) and surgical specimen (SS), in breast cancer, has long been a matter of discussion because of its influence on oncologic treatment choice. Particularly, human epidermal growth factor receptor 2 (Her2) status is quite important, because of the impact on breast cancer classification and target therapy. Many factors could influence the difference in biomarker status between samples, such as the technic itself, sample procedures and intratumoral heterogeneity. Neoadjuvant chemotherapy (NAC) can also contribute to this variation and should be taken into consideration. We report a clinical case of a 33-year-old man who was diagnosed with right breast cancer, initially Her2 negative in the CNB. Therefore, the NAC was completed without anti-Her2 treatment. Later, after the mastectomy, revision of the SS was found to be positive for Her2, after the silver in situ hybridization (SISH) technique. Consequently, the patient lost the chance of doing anti-Her2 therapy in the neoadjuvant setting, reducing his possibility to achieve a complete pathologic response and later jeopardizing his clinical outcome.

## Introduction

Breast cancer is one of the most common types of cancer worldwide and its prevalence continues to rise. Part of a good initial evaluation includes immunohistochemistry (IHC) determination of biomarker status, to decide the treatment strategy, particularly in the neoadjuvant setting [1-7]. Core needle biopsy (CNB) is always the choice for histologic diagnosis and the sample where biomarker evaluation is conducted [1,2,4-6,8,9]. The analysis of biomarker concordance between CNB and the surgical specimen (SS) is poorly documented but most of the studies suggest a good correlation between them [1-9]. This concordance seems to be great in all biomarkers, but human epidermal growth factor receptor 2 (Her2) is the one with the least conformity and both IHC and fluorescence in situ hybridization (FISH) technics play an important role [4]. Several factors, such as intratumoral heterogeneity, may influence the tumor biomarker profile [1]. Her2 expression is especially important because it impacts breast cancer classification and target therapy [6-10].

## Case presentation

A 33-year-old African man, natural from Cape Verde, with no relevant medical or family history was diagnosed with right breast cancer in April 2016, staged as cT4N1M0. CNB was compatible with invasive carcinoma, G2, estrogen receptor 90%; progesterone receptor 90%; Her2 2+ from IHC technic and negative when confirmed with silver in situ hybridization (SISH), Ki67=25%-30%. Therefore, the patient initiated neoadjuvant chemotherapy (NAC) with doxorubicin 60mg/m2 plus cyclophosphamide 600mg/m^2^, four cycles followed by 12 cycles of weekly paclitaxel 80mg/m^2^, from May to October 2016, with multiple delays due to neutropenia G2/G3.

**Figure 1:**
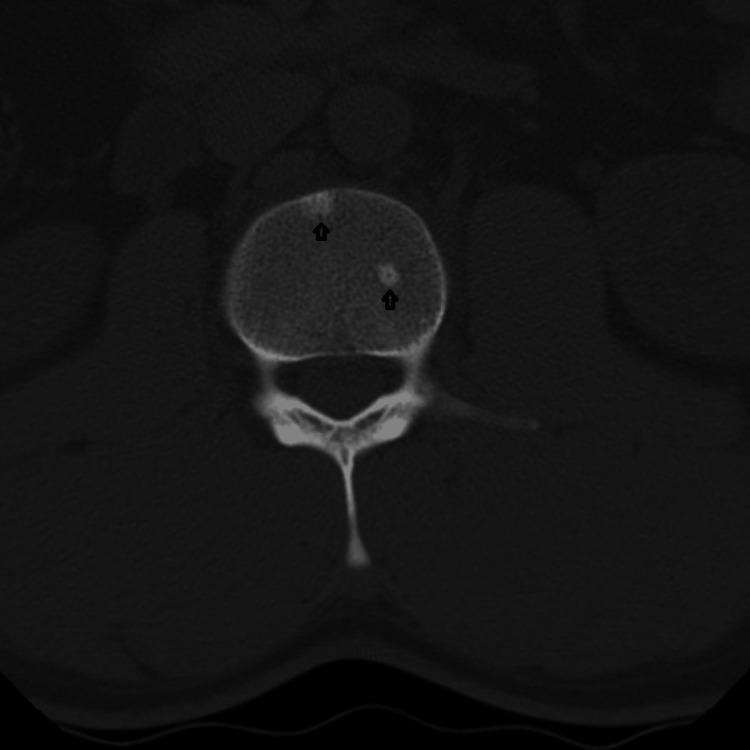
Axial section of L2 vertebra, with two osteoblastic metastases on plain CT scan

**Figure 2:**
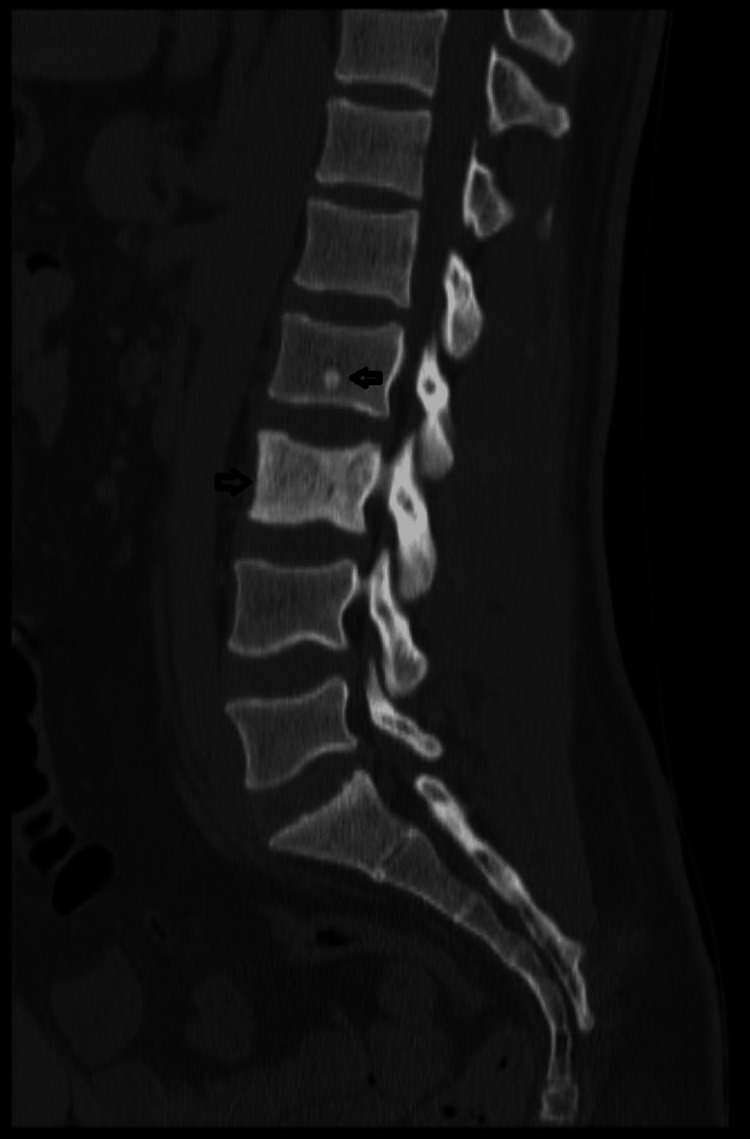
Sagittal section of the lumbosacral spine with metastases in L2 and L3 on plain CT scan

The patient then started to complain about low back pain and the lumbar computed tomography (CT) revealed bone metastasis in L3 and L2 (Figures 1, 2), with no other visceral metastasis confirmed by CT. Because of the limited bone metastatic disease, it was performed an oligometastatic approach, and the patient was submitted to a modified radical right mastectomy in November 2016. The pathologic result from the surgical resection was an invasive carcinoma, ypT4bN1a, Her2 positive using SISH. Both samples, from the biopsy and the mastectomy, were reviewed for the Her2 status and confirmed the discrepancy.

In the metastatic setting, the patient started treatment with trastuzumab 6mg/kg, every three weeks, daily tamoxifen 20mg, luteinizing hormone-releasing hormone (LHRH) analog monthly and bisphosphonates injections every six months, with the stable disease until January 2019. At this time, because of bone disease progression, the hormonal treatment was changed to letrozole, and maintained the rest of the strategy was until September 2021, when bone metastasis progression was evident in imaging exams. The patient then started second-line metastatic therapy with trastuzumab emtansine (TDM-1) 3.6mg/kg, every three weeks, and palliative radiotherapy, with disease stability until the last evaluation in June 2022.

## Discussion

Although most studies showed a good concordance between CNB and SS there are some constraints associated with the small number of samples and limited statistical power [3,5,8]. The literature refers a discordance in Her2 status in percentages that range from 8% to 18% [10]. Factors including the quantity of tissue available, the technic itself, the differences in the antibody used, laboratory experience, tumor sampling and fixation protocols may impact the results [1-5,8-10]. It is more common to upscore in CNB compared to SS because of the empirical knowledge that CNB samples fix better. Heat exposure, longer warm ischemia period and inadequate fixation of the SS may justify this difference [1,2,4,5,7].

Also, intratumoral heterogeneity may present as a challenge for clinicians and is responsible for 35-56% of biomarker discrepancy [1,2,4,6,7,9,10]. However, the chances that a tumor has both Her2 negative and positive regions are extremely low, especially after using in situ hybridization techniques [10]. Some studies suggest having a minimum of five samples in CNB and repeating biomarker status in SS when any biomarker is negative in the CNB, to prevent possible mistakes and amend decisions [2,6,9].

We cannot overlook the fact that neoadjuvant chemotherapy can change tumor characteristics over time because of selective pressure caused by antineoplastic drugs [3,7]. In fact, one study compared Her2 discrepancies in patients with and without NAC. The group who had undergone NAC had a greater discordance rate and factors that influenced Her2 discrepancy were large residual tumors and metastatic lymph nodes [6].

More important than the discrepancies itself is the impact on treatment choice and clinical implications. In this clinical case, the patient was not treated with anti-Her2 target therapy in the neoadjuvant setting, as it should. This shortened his chance of complete pathological response and furthermore has implications in his future clinical outcome [6,7]. The fact that this kind of discrepancy between samples is so rare, even more, with a positive result in the SS instead of CNB makes this a very unusual case.

## Conclusions

Biomarker concordance between CNB and SS seems to be reasonable; however, negative biomarkers in CNB should be repeated in the SS, particularly in patients who undergo NAC. Further longitudinal studies characterizing spatial biomarker distribution and identifying risk factors for intratumoral heterogeneity are needed to improve our understanding of whose patients may benefit from a more extensive workup and its impact on clinical outcomes.
